# Nail-Associated Body-Focused Repetitive Behaviors: Habit-Tic Nail Deformity, Onychophagia, and Onychotillomania

**DOI:** 10.7759/cureus.22818

**Published:** 2022-03-03

**Authors:** Philip R Cohen

**Affiliations:** 1 Dermatology, University of California, Davis Medical Center, Sacramento, USA

**Keywords:** tic, repetitive, pick, onychotillomania, onychophagia, nail, habit, body, bite, behavior

## Abstract

Habit-tic nail deformity, onychophagia (also referred to as nail biting) and onychotillomania (also referred to as nail picking) are body-focused repetitive behaviors that can involve the nails and periungual skin. Patients with habit-tic nail deformity are typically unaware that repeatedly using their nail, often the adjacent index finger, to rub the proximal nail fold and its underlying matrix - usually of one or both of their thumbnails - is the cause of the longitudinal depressed groove that extends along the entire the nail plate. Nail biters usually bite multiple nails - most commonly on the digits of the hands - and the patient is cognizant of their behavior. However, the term onychophagia is a misnomer and onychodaxia would be a more appropriate nomenclature. Nail pickers also often realize that their dystrophic nail results from using other nails or tools to pick, pull, or excessively manicure the affected nail. Individuals with habit-tic nail deformity or onychophagia or onychotillomania may concurrently have other repetitive behaviors involving the skin or the hair or both. Three patients with a nail-associated body-focused repetitive behavior are described who not only presented with dystrophy of their nails but also abnormalities of the adjacent nail apparatus: a 36-year-old woman with habit-tic nail deformity and dermatodaxia, a 64-year-old man with biting of both the nails and the skin, and a 63-year-old man with nail picking and skin picking. The nail dystrophy and concurrent skin biting or skin picking were not the issues that prompted the reported patients to seek evaluation by a physician; the body-focused repetitive behaviors of the nails and skin were incidental findings during their cutaneous examination. The management of nail-associated body-focused repetitive behavior may include non-pharmacologic treatments (such as physical modalities and behavior modifications) and/or pharmacologic agents. The reported woman with habit-tic nail deformity was willing to consider an attempt to modify her repetitive behavior by using paper tape as a physical modality to cover the area on her thumbs that she would unconsciously rub. However, similar to the men in this report with onychophagia and onychotillomania, many of the patients with nail-focused repetitive behaviors do not want to initiate any interventions that might decrease or eliminate their nail condition.

## Introduction

Nail-associated body-focused repetitive behaviors include habit-tic nail deformity, onychophagia, and onychotillomania. Habit-tic nail deformity presents as a longitudinal depressed groove involving the entire nail plate. It typically affects either one or both thumbnails [1,2].

Onychophagia describes nail biting. It often involves multiple nails. Although it more commonly occurs on the fingernails and thumbnails, it can involve the nails of the toes or the nails of digits on both the upper and lower extremities [3,4].

Onychotillomania is a disorder of nail picking. However, several investigators consider this nail dystrophy to not only include the nail plate but also the underlying and surrounding nail unit. Patients with this condition create an onychodystrophy of their fingernails and/or toenails by using their nails or other tools to pick, pull, or excessively manipulate the affected nails [5,6]. 

Three patients with either habit-tic nail deformity, onychophagia, or onychotillomania are described. The features of nail-associated body-focused repetitive behavior are reviewed. In addition, possible interventions for these conditions are summarized.

## Case presentation

Case 1

A 36-year-old woman had noticed an itchy and painful lesion on her left arm that had increased in size during the past three years. Her past medical history was only significant for surgery to correct a cerebral arteriovenous malformation 17 years earlier. The only medication she was receiving was levetiracetam, for seizure prophylaxis, 750 milligrams twice daily.

**Figure 1:**
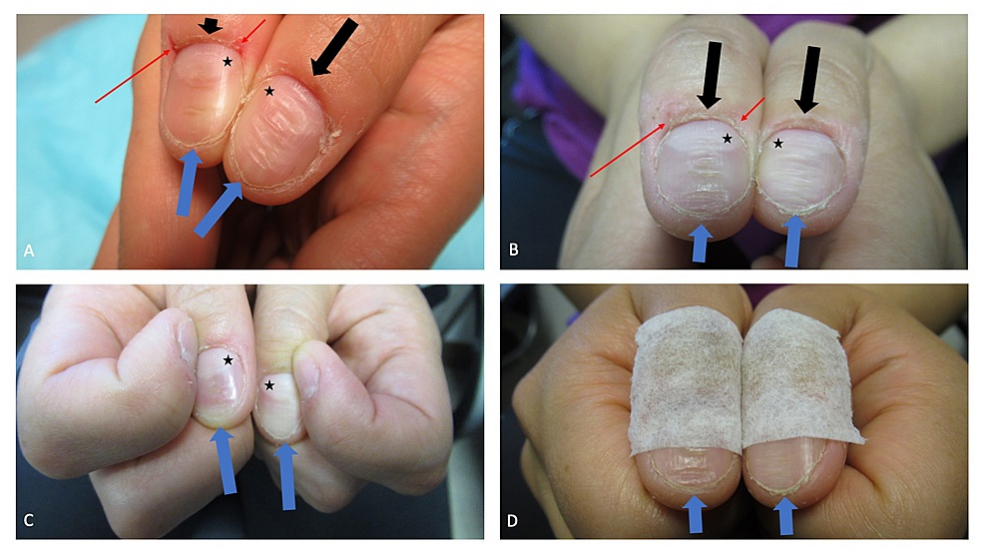
Habit-tic nail deformity on both thumbs At her initial visit, the thumbs of a 36-year-old woman both showed the manifestations of bilateral habit-tic nail deformity characterized by absent cuticles, abraded proximal nailfolds, enlarged lunula (black asterisks), and a furrow consisting of transverse fissures that extended from the proximal nailfold (black arrows) to the distal tip of the nail (blue arrows); in addition, both edges of the proximal nailfold of her right thumb had a fissure and an erosion (red arrows) caused by repetitive skin biting (A). Two weeks later, after applying mupirocin 2% ointment three times daily, the fissure and erosion had resolved but the linear nail plate grooves persisted (B). Repeated rubbing of the proximal nail fold of the thumb with the adjacent index fingernail created the nail dystrophy (C). As an intervention to diminish her nail-associated repetitive behavior, hypoallergenic paper tape was applied from the interphalangeal joints of the thumb to the middle of the nail plate (D).

Cutaneous examination showed a tender 6 x 6-millimeter hyperpigmented dermal nodule on the deltoid region of her left arm. Her thumbs also showed abnormalities of the nails and surrounding skin. There were fissures and erosions on the right thumb at both edges of the proximal nailfold. Both thumbs also showed the same changes; the cuticles were absent, the proximal nailfolds were abraded, the lunula was enlarged, and there was a groove composed of horizontal fissures that extended from the proximal nailfold to the distal tip of the nail (Figure 1A).

A punch biopsy of the left arm lesion was performed and the specimen was evaluated; a diagnosis of a benign dermatofibroma was established. Additional history revealed that the right proximal nail fold lesions were secondary to the patient repetitively biting these areas; mupirocin 2% ointment, three times daily, was initiated to treat the dermatodaxia lesions. The linear grooves on the thumb nails were a habit-tic nail deformity.

She returned for evaluation two weeks later. The biopsy site on her left arm had healed; although residual dermatofibroma was present, the area was no longer painful, and additional treatment was not necessary. However, there was pruritus and erythema that corresponded to the adhesive from the band aids she had used, establishing a diagnosis of allergic contact dermatitis to the band aid adhesive.

At her follow-up visit, the fissure and erosion on the proximal nail fold of her right thumb had resolved; however, the linear grooves on the nail plates of both thumbs persisted (Figure 1B). She was unaware of their etiology, yet, after inquiry, readily admitted that she would repeatedly use her index fingernails to rub the central portion of the ipsilateral proximal nail fold of the thumb (Figure 1C). The cause of the nail plate dystrophy - intermittent, yet chronic, injury to the nail matrix beneath the proximal nail fold that she would rub - was explained to the patient; thereafter, she agreed to try applying hypoallergenic paper tape (since she was allergic to the adhesive on band aids) from the interphalangeal joints of the thumb to the middle of the nail plate as a deterrent to rubbing the nail fold with her index finger (Figure 1D).

Case 2

A 64-year-old man presented for a skin check. He had a medical history of gastroesophageal reflux disease, hypercholesterolemia, hypertension, obstructive sleep apnea and prediabetes; his daily medications included atorvastatin, ezetimibe, lansoprazole, and losartan. He also had a history of actinic keratoses and three basal cell carcinomas.

A complete cutaneous examination was performed. Nine erythematous scaling plaques, consistent with actinic keratoses, were found on the face and legs; these were treated with cryotherapy using liquid nitrogen. A 5 x 5-millimeter red plaque was noted on the left side of his cutaneous upper lip; a biopsy was performed which demonstrated a squamous cell carcinoma in situ that was subsequently excised using microscopically controlled surgery to confirm complete removal of the tumor.

**Figure 2:**
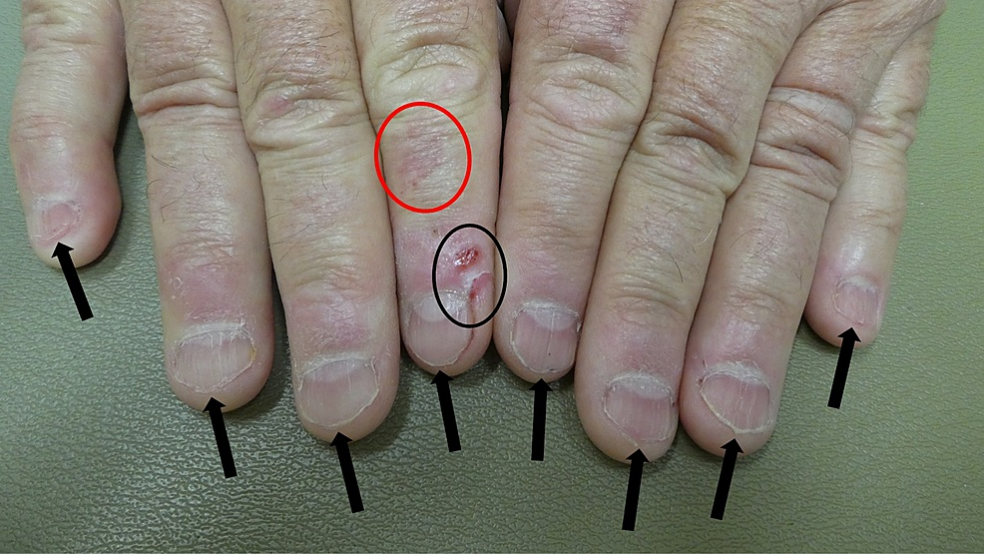
Onychophagia of the fingernails A 64-year-old man had short fingernails on both hands. The distal edge (black arrows) of each nail was irregular and there was lamellar onychoschizia (horizontal splitting) of the distal margin of the nail plate of the right fifth digit; these changes were from repetitive nail biting. An erosion and skin tear were also present on the distal right index finger (within the black oval); these changes were caused by skin biting. A congenital hemangioma, appearing as a red patch, was also present on his right index finger (within the red oval).

**Figure 3:**
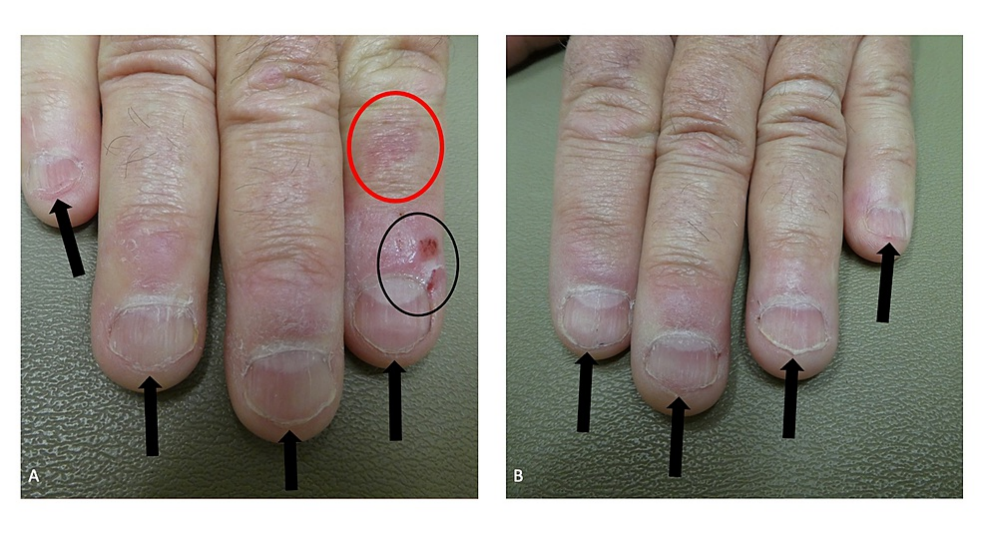
Nail biting affecting the fingers of the right and left hand A closer view of the fingernails on the right (A) and left (B) hands of a man with onychophagia and dermatodaxia. Short fingernails with irregular edges (black arrows) are caused by recurrent biting of the nails; there is horizontal splitting of the distal nail plate of the right fifth digit. Skin biting of the right index finger resulted in an erosion and tear (within the black oval); these lesions completely healed after being treated three times daily with mupirocin 2% ointment. A hemangioma, that had been present since birth, is also present on the right index finger (within the red oval).

**Figure 4:**
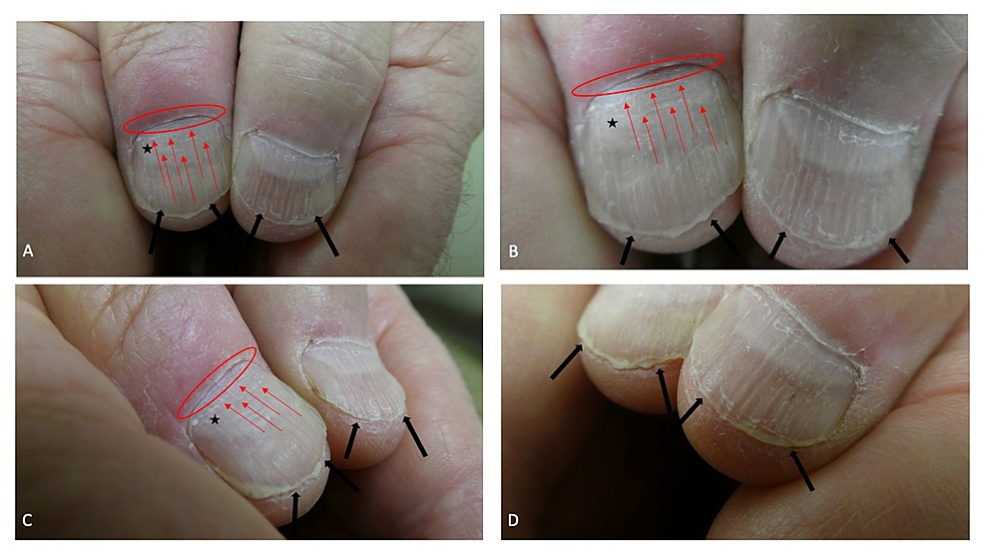
Onychophagia of the thumbnails Distant (A), closer (B), right sided (C), and left sided (D) views of the thumbs of a man who repetitively bites his nails. In addition to age-related longitudinal ridges and furrows, the nail plates are short and their edges are irregular (black arrows); there was also lamellar onychoschizia (horizontal splitting) of the distal right thumbnail. The cuticle on his right thumb is absent (red oval); this was caused by periodically rubbing away the cuticle with his left thumb. In addition, the left thumb has an enlarged of the lunula (black asterisk showing the macrolunula) and two transverse ridges across the proximal nail plate (red arrows showing the Beau’s lines) that correspond to each episode of the cuticle being removed by rubbing.

Cutaneous examination of his hands showed a red patch on his right index finger between the interphalangeal joints (Figures 2, 3); this had been present since birth and was a congenital hemangioma. All his fingernails and both thumbnails were short (Figures 2, 3, 4); also, the distal margins of the nail plates were irregular and there was horizontal splitting of the distal right thumbnail and fifth digit nail plate. He subsequently admitted that he regularly bit the nails of his fingers and thumbs; he did not want to consider any intervention to stop his onychophagia.

The cuticle on his right thumb was absent, the lunula on that thumb was enlarged, and there were two horizontal lines across the proximal nail plate (Figure 4). He mentioned that he would use his left thumb to periodically rub away the cuticle on his right thumb. The transverse lines on the nail plate correspond to each episode of cuticle removal.

The radial side of his distal right index finger had an erosion; also, on the same side of the digit, there was a skin tear on the corner of the proximal nail fold (Figures 2, 3). He confirmed that he had bitten the skin at both sites. Both dermatodaxia lesions were treated with mupirocin 2% ointment that was applied three times daily; they completely healed. 

Case 3

A 63-year-old man presented for a skin check. He had a medical history of chronic hepatitis C, depression, hyperlipidemia, hypertension, irritable bowel syndrome and migraine headaches; his daily medications included clonazepam, lubiprostone, nortriptyline, propranolol, simvastatin, topiramate, and venlafaxine. He also had a history of actinic keratoses.

A complete cutaneous examination was performed. Thirty-eight erythematous scaling plaques, consistent with actinic keratoses, were found on the face and legs; some of these were treated with cryotherapy using liquid nitrogen and the remainder were treated with topical fluorouracil 5% cream. An 8 x 8-millimeter ulcerated nodule was noted on the left helical rim; a biopsy was performed which demonstrated an infiltrative basal cell carcinoma which was subsequently excised using microscopically controlled surgery.

**Figure 5:**
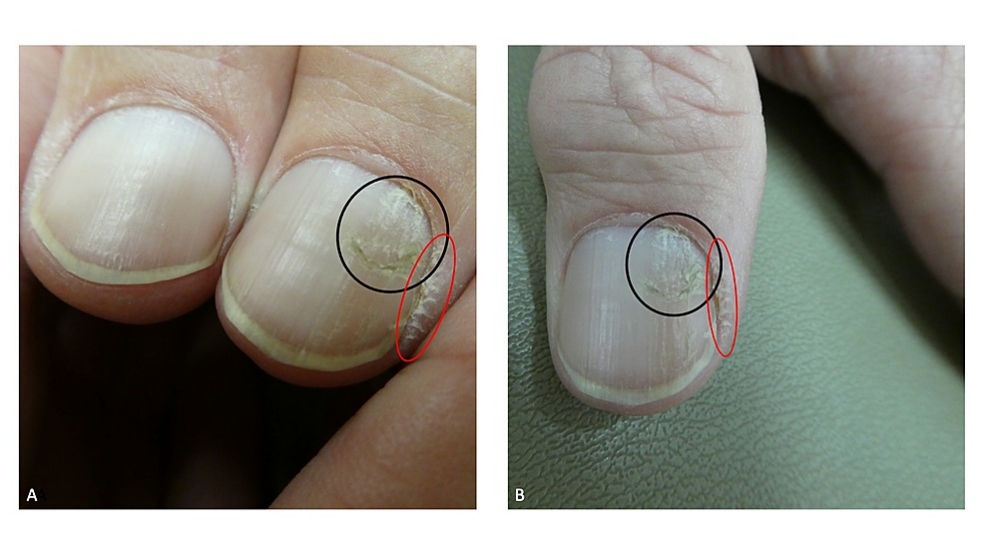
Onychotillomania of the left thumbnail Left side (A) and superior (B) views of the nail picking (within the black oval) involving the left thumbnail of a 63-year-old man; a subtle longitudinal furrow is observed both proximal and distal to the horizontal fissure. He is also a skin picker; the cuticle is absent from the hypertrophic and ragged lateral nail fold, on the ulnar side of his thumb (within the red oval), which also shows scaling and superficial fissures.

Cutaneous examination of his hands showed changes of not only the nail plate, but also the nail fold of the left thumb (Figure 5). There was a horizontal fissure, located 6 millimeters from the proximal nail fold, with a subtle longitudinal furrow both proximal and distal to the dystrophic nail plate. The lateral nail fold, on the ulnar side, was also altered; it was ragged, hypertrophic, and the skin surface demonstrated superficial fissures and scaling. The cuticle was also absent in the affected areas.

**Figure 6:**
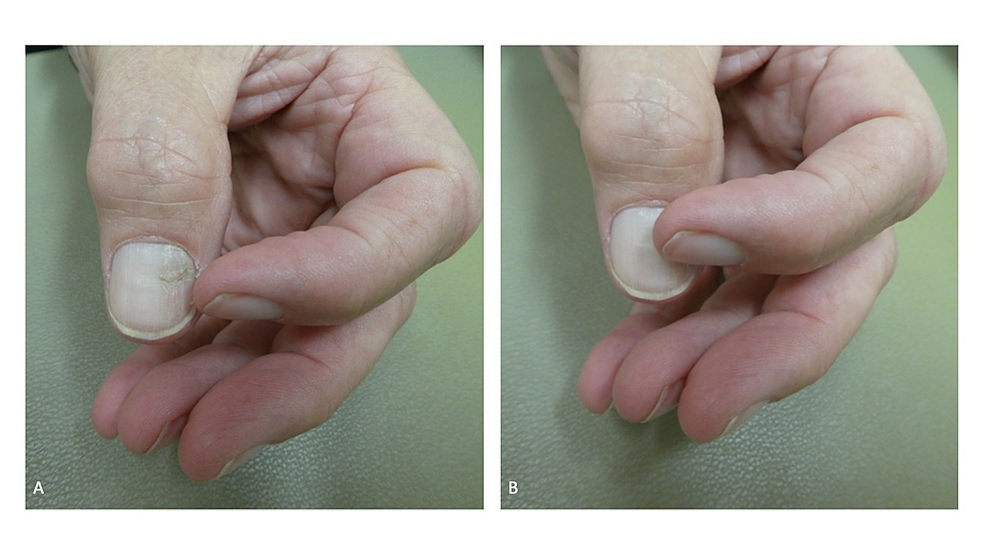
Nail picking of the left thumbnail using the fingernail of the ipsilateral second digit The alteration of the lateral nailfold and the dystrophy of the nail plate of the patient’s left thumb were manifestations of his skin picking and onychotillomania. The man demonstrated how he would use the nail of the adjacent left index finger to not only pick the lateral nail fold (A) but also the nail plate (B) of his left thumb.

Additional inquiry regarding the cause of the findings on his left thumb was conducted. The patient explained that he regularly used the lateral edge of the adjacent index fingernail to rub and pick at the nail plate and nail fold of the left thumb; correlation of the clinical history and morphologic presentation established the diagnoses of onychotillomania and skin picking. He also eagerly demonstrated how he performed these activities and did not want to initiate any intervention to stop the behavior (Figure 6).

## Discussion

A revised classification of obsessive-compulsive and related disorders was adopted in 2013 when the Fifth Edition of the American Psychiatric Association publication of Diagnostic and Statistical Manual of Mental Disorders (DSM5) was published. Subsequently, beginning in 2022, this classification was also incorporated by the World Health Organization’s International Classification of Diseases and Related Health Problems, Eleventh Revision (ICD-11) in the chapter on Mental and Behavioral Disorders. Indeed, in addition to lip chewing, several nail disorders (such as habit-tic nail deformity, onychophagia, and onychotillomania) were included in the section referred to as unspecified body-focused repetitive behaviors [7,8].

Nail-associated body-focused repetitive behaviors occur not only as a solitary obsessive-compulsive condition but also concurrently with other body-focused repetitive behaviors of the hair and skin [1,3,9,10]. The Wisconsin Psychocutaneous Clinic evaluated referring and final diagnoses of 808 patients, several of whom had more than one final diagnosis, during a period of nearly 16 years. A maximum of 14 individuals had onychotillomania; of these individuals, 11 patients were also skin pickers and one patient picked both their skin and hair [11].

All three of the patients in this report have more than one body-focused repetitive behaviors. The woman with habit-tic nail deformity also had dermatodaxia and would bite her nail folds. The man with onychophagia would not only rub his cuticles but also bite the skin of his nail fold. And the man with onychotillomania of his left thumb was also a skin picker of the nail fold on the same digit.

**Figure 7:**
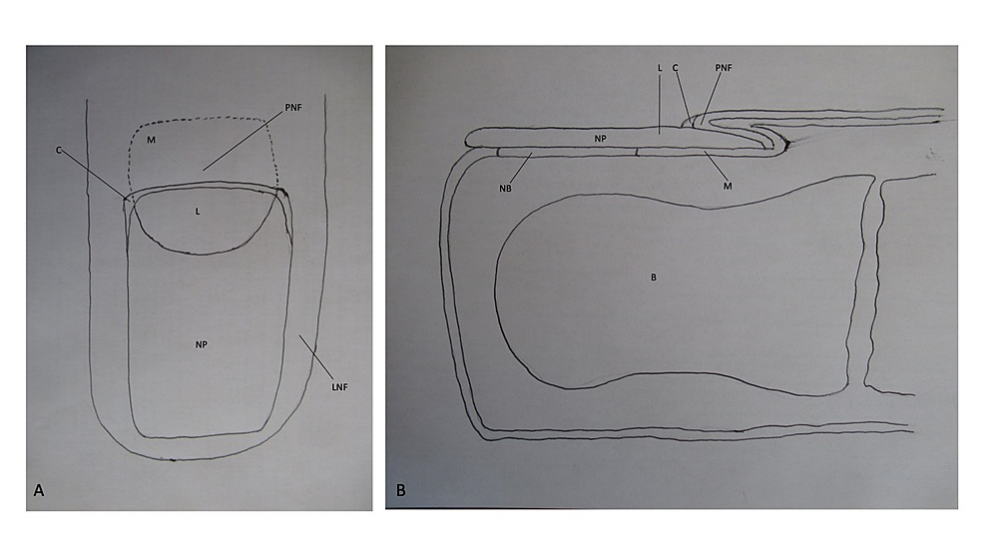
Nail unit anatomy Superior view (A) and side view (B) of a distal digit show the structures that comprise the nail unit; the nail plate (NP) is in close proximity to the underlying bone (B). The nail plate extends from the proximal nail fold (PNF); the distal portion of the nail matrix (M), that is not covered by the proximal nail fold, appears white and is referred to as the lunula (L). The distal nail plate, which overlies the nail bed and normally extends to the tip of the digit, appears pink. The proximal nail fold and the lateral nail fold (LNF) comprise the skin that surrounds the nail plate. The proximal nail fold covers the proximal nail matrix (whose boundary is designated by the black dotted line); the nail plate originates from the proximal nail matrix. The space between the proximal nail fold and the nail plate is sealed by the cuticle (C). The drawings were created by the author.

An awareness of nail unit anatomy may enable a greater appreciation and understanding of nail-associated body-focused repetitive behaviors (Figure 7). The nail plate normally extends from the proximal nail fold to the tip of the digit. The lunula is the white-appearing proximal portion of the nail plate; it covers the distal portion of the nail matrix. The distal nail plate, which covers the nail bed, appears pink.

The nail plate is surrounded by skin: the proximal nail fold and the lateral nail fold. The cuticle normally seals the space between the proximal nail fold and the nail plate. The nail plate originates from the proximal matrix which is located beneath the proximal nail fold.

Habit-tic nail deformity is a distinctive condition. It can occur in children; however, it is commonly observed in adults. The affected individual usually performs the nail-focused repetitive behavior subconsciously and is therefore unaware that they are causing their nail dystrophy [1,2,12].

Habit-tic nail deformity can occur on any digit; however, it most commonly appears on one or both thumbnails. It develops from the repeated manipulation (by rubbing or picking) of the affected digit’s cuticle, proximal nail fold, or both. The consequence of the habitual contact results in trauma to the underlying nail matrix and manifests as a dystrophic nail [1,2,12].

The habit-tic nail deformity has a characteristic presentation: a central longitudinal furrow with transverse parallel ridges that extend from the proximal nail fold to the distal end of the nail plate. Similar to the woman in this report, enlargement of the lunula (referred to as macrolunula) is often observed on the affected digit. Complications of habit-tic nail deformity may include frictional melanonychia, infection, and permanent nail dystrophy [1,2,12].

The main differential diagnosis of habit-tic nail deformity is median canaliform dystrophy. The latter condition presents as a midline nail plate split with multiple, oblique and proximal outward ridges from the central split nail in a fir-tree pattern; in contrast to habit-tic nail deformity, the cuticle is intact in median canaliform dystrophy. The etiology of median canaliform dystrophy remains to be established [1,2,12].

The initial intervention in the management of habit-tic nail deformity is explaining the pathogenesis of the condition to the patient who typically is unaware that they are non-intentionally causing their nail dystrophy. Once an explanation has been provided, the patient is able to associate that their habitual manipulation of the affected digit is causing the observed nail plate abnormality. However, even after the patient understands the etiology of their nail disorder, they often do not want to initiate any actions to stop the repetitive behavior [1,12].

Once the patient with a habit-tic nail deformity stops manipulating the skin or cuticle overlying the affected nail matrix, the nail dystrophy often spontaneously resolves. Treatment of habit-tic nail deformity can be non-pharmacologic or pharmacologic. For example, a topical ointment such as petroleum jelly can be gently massaged over the affected area several times daily [1,2,12].

Similar to the woman in this report, a physical barrier can be used to prevent the patient from rubbing or picking at the proximal nail fold or cuticle. A band aid is often used; however, hypoallergenic paper tape was used in the reported patient since she had allergic contact dermatitis to band aid adhesive. Since patients keep the occlusive dressing on for several hours, the dressing should be applied in a longitudinal direction that only partially covers the digit (such as from the skin proximal to the nail fold distally to the nail plate) so that the digit is not completely encased and constricted by the dressing [1,2,12]. In addition, similar to dermatodaxia, thermoplastic protective gloves can be created; these have been referred to as protecting little and adolescent youth (PLAY) Hands [10].

Other non-pharmacologic interventions include applying either cyanoacrylate adhesive (which is used in super glue) or benzethonium chloride (which is used as an antiseptic) to the nail (where the proximal nail fold is present) to artificially recreate the cuticle and prevent trauma to the underlying nail matrix. Cyanoacrylate has been associated with allergic contact dermatitis; therefore, benzethonium chloride may have less potential for therapy-associated adverse events [1,2,12]. Cognitive behavioral therapy or habit reversal therapy are other non-pharmacologic interventions that may also be considered [1,2,12-14].

Cognitive behavioral therapy can have a pivotal role in treating psychodermatoses and has emerged as one of the first-line psychotherapies for not only habit-tic nail deformity but also other nail-associated body-focused repetitive behaviors such as onychophagia and onychotillomania. Cognitive behavioral therapy, a problem-oriented psychotherapy, attempts to modify the thought patterns (reflecting the patient’s cognitive conceptions) and actions (reflecting the patient’s behavioral activities) that are currently dysfunctional and resulting in not only injury to their nails, but also psychological distress. This approach to therapy involves having the patient examine and address their inaccurate concepts regarding the cause of their nail disorder and thereby replacing the improper and distorted thoughts with more appropriate and valid dogma so that they are then able to modify and subsequently decrease or eliminate their behavior [14,15].

Cognitive behavioral therapy sessions, between the therapist and the patient, usually occur weekly for 12 to 20 weeks. During this period, preliminary improvement may be observed; however, sustained long-term success often requires more than 20 treatments. Patients with body-focused repetitive behavior who also have other psychiatric disorders, such as anxiety disorder, obsessive-compulsive and related disorders, and somatic symptom disorders, may most benefit from cognitive behavioral therapy. Indeed, the investigators of an anxious seven-year-old girl with habit-tic nail deformity and concurrent trichotillomania commented that educating the child’s parents regarding the possibility of cognitive behavioral therapy for the management of her anxiety was particularly relevant for their patient [1,14,15].

Pharmacologic therapy may be helpful in patients whose habit-tic nail deformity is severe or persistent or in those individuals with concurrent psychiatric conditions. Agents that have been used include selective serotonin reuptake inhibitors, N-acetylcysteine, tricyclic antidepressants, and antipsychotic drugs. However, the results with these medications are variable and the therapies may be associated with drug-associated side effects [1,2,12,15].

Onychophagia is a common disorder with a prevalence ranging from 20 to 30% of people. More commonly, it is referred to as nail biting. Onychophagia is defined as biting on the nails (of either the digits of the hand or feet or both) with the teeth after putting one or more fingers, thumbs, and/or toes into the mouth [3,4,12,16]. 

However, the term onychophagia is a misnomer. Onychophagia implies not only biting the nail, but also consumption of the nail plate. Although some of the people who bite their nails may also chew the nail plate, very few of these individuals eat the nail that they have bitten [12]. 

A related issue of terminology was evaluated by investigators regarding the inappropriate use of the term dermatophagia to refer to skin biting since the patient typically does not eat the skin that they have bitten. The researchers searched Greek dictionaries and discovered that ‘dermato’ refers to ‘of the skin’ and ‘daxia’ refers to ‘the act of biting’; hence, they recommended that skin biting be designated as dermatodaxia [17]. Therefore, similar to the nomenclature for skin biting, nail biting should more appropriately be termed onychodaxia; however, onychophagia, which is currently the established term for this condition in the medical lexicon, is used in this paper.

Onychophagia is rarely observed in children less than three years old; in a study of pediatric nail biting, the median onset age was five years old. The prevalence reached a peak in adolescence and decreased after puberty; indeed, the behavior is discontinued by many children as they become older. However, adult-onset onychophagia can occur [3,4,12,16].

The condition may be associated with genetic factors. Monozygotic twins have a higher concordance rate of nail biting as compared to dizygotic twins. Also, onychophagia occurs more often in individuals who have a parent that bites their nails; hence, after diagnosing onychophagia, particularly in a pediatric patient, it might be reasonable to check the nails of other family members for nail biting [3,4,12,16].

Onychophagia may be associated with concurrent psychiatric conditions. In pediatric patients, 88% (92 or 104 children) only bit their fingernails compared to 12% (12 of 104 children) who concurrently bit both their fingernails and toenails; indeed, psychiatric disorders were more common in children who bit both fingernails and toenails as compared to those who only bit their fingernails [16]. Although some investigators have associated stress and anxiety with nail biting, several studies do not support this hypothesis [3,4,12]. 

Onychophagia has been observed in children with attention deficit hypersensitivity disorder [3,16]. One group of researchers also noted nail biting to occur in patients with oppositional defiant disorder and separation anxiety disorder. In addition, other conditions present in patients with onychophagia include enuresis, major depressive disorder, mental retardation, and pervasive developmental disorder [3,12,16].

Nail biting may occur in patients with an obsessive-compulsive disorder. Also, other body-focused repetitive behaviors have been observed in patients with onychophagia [3,12,18]. Similar to the patient in this report, skin picking may occur [9]. 

More than half of the patients in one study bit their nails at least once daily [16]. Typically, multiple nails, often with symmetric presentation, are involved. The nails are abnormally short and the distal edges are uneven, with or without horizontal splitting of the nail plate (which is referred to as lamellar onychoschizia); in severe cases, biting occurs past the nail bed, or the nail is absent or both. The cuticles may be ragged or absent and the nail folds may also be dystrophic; although some investigators elect to consider these to be changes of onychophagia, other researchers more appropriately classify these findings as a concurrent occurrence of other body-focused repetitive disorders such as skin picking and skin biting [4,10,12,19].

Complications of onychophagia that affect the nail unit can include either bacterial or fungal infection resulting in acute paronychia or chronic paronychia or both. Other infections can affect the bone (resulting in osteomyelitis) or be caused by viral pathogens (such as herpetic whitlow from herpes simplex virus or subungual verruca vulgaris from human papillomavirus). In addition, nail and nail fold complications from nail biting occur from trauma to the nail bed and/or nail matrix (such as longer nail plates from increased rate of nail growth, longitudinal melanonychia, shorter nail plates from loss of the nail bed, splinter hemorrhages, and rarely an intraosseous epidermoid cyst) [4,12].

Onychophagia can also have oral and dental complications. Temporomandibular joint syndrome has been associated with nail biting. In addition, people who bite their nails have been shown to have higher oral bacteria carriage (of *Escherichia coli* and* Enterobacter *species) and bacterial colonization (of Methicillin-resistant* Staphylococcus epidermidis*); nail-biters also can develop apical root resorption, crowding and rotation of incisors, croup-like presentation from aspiration, gingival abscesses, gingival swelling, infection (caused by embedment of the nail plate), and malocclusion of teeth [4].

The management of onychophagia includes observation and prevention of secondary complications in patients who do want to initiate treatment. Alternatively, there are several approaches that do not involve oral medications. However, for some individuals, pharmacologic agents may be helpful [3,4,9,10,12,13,15,16,19,20].

The most common non-pharmacologic treatments for nail biting include punishment and habit reversal therapy [4]. Punishment attempts to stop the repetitive behavior by reprimanding the individual. However, this form of intervention has not only been unsuccessful in eliminating onychophagia (since it was not demonstrated to be more effective than placebo), but also may increase the patient’s nail biting (since the individual gains attention by biting their nails) [3,4].

Habit reversal therapy has resulted in decreasing the episodes of nail biting for individuals treated with this modality. It can be used as monotherapy or in combination with other treatments, such as pharmacotherapy with selective serotonin reuptake inhibitors, particularly in patients who concurrently have obsessive-compulsive disorder. This therapy includes a minimum of three components: awareness training, competing response training, and social support. Some researchers also include two additional components: stimulus control training and relaxing training [3,4,13,15,20].

Awareness training requires the patient to acknowledge that they are cognizant of their nail biting disorder and its consequences. It also encompasses the patient being aware of specific antecedent warning signs or trigger activities that occur prior to biting their nails. Stimulus control training involves the patient avoiding situations that subsequently result in onychophagia. In addition, relaxation training can diminish the associated urge and stress that can also be a cause for their nail-associated repetitive behavior [13,15,20].

Competing response training incorporates the development of a distracting behavior - such as holding an object (for example, a pencil or a toy or a small ball) or writing, or drawing - that the patient can perform and thereby making it physically impossible for them to bite their nails until the desire to do so has subsided. Finally, social support entails the recruitment of a person whom the patient can rely on to provide not only praise and encouragement when they successfully engage in competing responses, but also objective feedback and advise when they are not able to correctly or adequately perform the response necessary to compete with their onychophagia urge and have allowed the repetitive nail biting behavior to occur [13,15,20].

Other non-pharmacologic treatments of onychophagia are chewing gum, cognitive behavioral therapy, cue-controlled relaxation, hypnosis, positive reinforcement, progressive muscle relaxation, psychotherapy, stimulus control training, object manipulation therapy, and occlusive barrier therapy [3,4,13,20]. Cognitive behavioral therapy assists the patient to change the incorrect or distorted conceptions that they have regarding their behavior. For example, a person who believes that biting their nails is helpful to them by initially providing a soothing effect may need to be challenged to understand the subsequent hours and days of shame from the disfigurement that occurs associated with the resulting nail dystrophy [3,16,20].

Object manipulation therapy requires the patient to manipulate an object when they have the urge to bite their nails. This can be a successful treatment for onychophagia; however, in a study comparing object manipulation to habit reversal therapy, object manipulation was less effective at three months follow up and had a higher dropout rate. Also, similar to using PLAY Hands for patients with skin biting, nail biting can be prevented by using thermoplastic protective finger wear for the involved digits or a complete glove if necessary [4,10].

Aversion therapy has also been used to onychophagia. However, aversion therapy is not unique to human who bite and chew their nails; it has also been used for animals, such as dogs, that excessively lick and/or chew on their elbows or paws. For these canines, in addition to establishing the etiology of the behavior and preventing access to the behavior with booties or bandages for the extremities and an Elizabethan collar that does not allow oral contact with the affected site, symptomatic treatment with interrupters that impede the behavior and aversive conditioners that create an unpleasant association with the behavior are utilized. For example, solutions or gels containing bitter yet non-toxic compounds are either sprayed or topically applied to the bootie or bandage to passively punish the dog each time it licks or chews its paw; the aversive conditions are effective in decreasing the frequency of the paw-focused repetitive behavior [3,4,20].

Similar to canines, aversion therapy for an individual with onychophagia can be accomplished by applying a bitter tasting lacquer to the nails in an attempt to deter the patient from nail biting since they will want to avoid the bad tasting substance. However, this is usually not an effective treatment. Therefore, a milder aversion technique is for the patient to apply a non-removable reminder, such as a rubber band on the wrist; the band serves as a reminder of behavior modification so that the individual does not bite their nails [3,4,20].

Combination of therapies (such as habit-reversal therapy and stimulus control) can also be used to treat onychophagia. In addition, weekly follow-up with a manicure that includes the application of a topical nail polish as a nonremovable reminder can enhance patient compliance. And, for more difficult patients who bite their nails, motivation for treatment can be enhanced by daily occlusion of only a single nail for two weeks so that they can observe clinical improvement and be motivated to stop biting the other affected nails [20]. 

Several agents have been evaluated for the pharmacologic management of onychophagia. These include N-acetylcysteine, dopamine agonists, lithium, selective serotonin reuptake inhibitors, and tricyclic antidepressants; common potential adverse effects that can be associated with the latter group of drugs include blurred vision, constipation, drowsiness, orthostatic hypotension, urinary retention, and xerostomia [4,14,15]. With regard to antidepressants, clomipramine was more efficacious than desipramine [12].

First-line drug treatment for nail biting is selective serotonin reuptake inhibitors. However, in onychophagia patients who also bipolar disorder, occasional individuals have developed treatment-induced mania. Yet, in bipolar disorder patients who are severe nail biters, lithium has been shown to be effective. And, in onychophagia patients with concurrent depression, the norepinephrine-dopamine reuptake inhibitor bupropion not only improved an individual’s depression but also the coexisting nail biting [12].

N-acetylcysteine is safer than the other systemic medications that have been used to treat onychophagia. Although it is effective in the management of nail biting, it was not demonstrated to be superior to placebo [4,12,16]. However, other investigators described the successful treatment of several patients who bite their nails with 1200 to 2400 milligram daily of N-acetylcysteine [20].

Onychotillomania describes recurrent picking of the nails [5,12,19,20]. However, several investigators have broadened the definition of this condition to include repetitive picking, pulling, and excessive manicuring of the entire nail unit; hence, they consider habit-tic nail deformity to be a variant of onychotillomania [5,6,18]. Yet, some of these same researchers have either commented that habit-tic nail deformity is a distinct behavior disorder of the nail or published management interventions specifically for patients with habit-tic nail deformity [2,5].

Onychotillomania is an uncommon disorder [12]. Individuals with onychotillomania frequently also have other body-focused repetitive behaviors (similar to the man in this report who was also a skin picker) and/or psychiatric conditions such as delusions of infestation, depression, hypochondriacal delusions, obsessive-compulsive disorder, phobias, and psychosis [6,18]. In addition, onychotillomania is a feature of Smith-Magenis syndrome; this is a neurodevelopmental disorder, most commonly associated with a mutation on the 17^th^ chromosome, that is also characterized by behavioral problems, facial dysmorphism (presenting with coarse features), intellectual deficits, peripheral neuropathy, short stature, sleep disorders, and speech delay [5,6].

Onychotillomania occurs 1.5 times more frequently in men than women. The average age of diagnosis is 47.5 years. However, nail picking typically begins in children or adolescents [5].

A review of the literature, which included 24 patients, showed that more than half of individuals with onychotillomania (54%, 13 patients) pick multiple fingernails on one or both hands; thereafter, in order of decreasing frequency, nail picking included both fingernails and toenails (16.7%, four patients), bilateral thumbs (12.5%, three patients), only toenails (12.5%, three patients), and only one fingernail (8.3%, two patients). The majority of individuals with onychotillomania (71.4%) would either pick their nails several times a day or all day as compared to the remaining 28.6% of patients who only pick their nails several times each week. Although most individuals with onychotillomania use their fingernails, predominantly those of the thumbs and index fingers, to pick at their other nails, other patients use tools to accomplish the task; these may include, but are not limited to, knives, scissors, toothpicks, and razor blades [6].

Nail picking morphologically presents as a dystrophic, or absent in severely affected patients, nail. The affected nail can be asymmetric and the architecture of the nail plate may be bizarre, similar to that of the patient in this report. Since the nail picker may also have other repetitive behaviors (such as habit-tic nail deformity, onychophagia, skin biting, and/or skin picking) additional findings may include central furrow with transverse parallel ridges, cuticle loss, macrolunula, melanonychia, nail bed exposure, nail fold hyperpigmentation, nail fold and hyponychium trauma (presenting as acute and/or chronic paronychia, erythema, erosions, and excoriations), nail plate atrophy or absence, nail plate shortening, pterygium formation, and subungual hematoma. In addition to bacterial and candida infections of the nail fold, onychotillomania can make the affected digit susceptible to viral infections (such as herpetic whitlow caused by herpes simplex virus, verruca vulgaris caused by human papillomavirus, and molluscum contagiosum caused by pox virus) [5,6,19].

In contrast to habit-tic nail deformity, patients with onychotillomania are usually aware of their self-aggressive, nail-focused, repetitive behavior [12]. However, management of these individuals may be unsuccessful since many of the patients deny that they are nail pickers; in addition, even individuals who readily admit to picking their nails, such as the man in this report, do not want to initiate any interventions to modify their behavior [5]. Some individuals with onychotillomania experience relief of existing tension after picking their nails; indeed, that person may have a concurrent obsessive-compulsive disorder and possibly benefit from a psychiatric evaluation [6].

Approaches to treatment of onychotillomania include pharmacotherapy and non-medication interventions. The latter include cognitive behavioral therapy, habit-reversal therapy, stimulus control procedures and occlusive barriers. Indeed, some researchers recommend combining psychotherapies [5,6,12-15,19,20].

Cognitive behavioral therapy, by replacing incorrect thoughts with accurate facts, strives to enable the patient with onychotillomania to adjust and possibly cease the repetitive and destructive behavior that they are performing to their nails. For example, a management intervention with cognitive behavioral therapy can be utilized for a patient with nail picking who is convinced that they are able to only pick their nails for a limited period of time and can then stop the behavior. In this scenario, the therapist can challenge the onychotillomania patient by requesting them to objectively document a situation in which they were able to restrict themselves to less than five minutes of nail picking [15,20].

Occlusive barriers attempt to protect the nails from external trauma. In addition, they serve as a reminder to avert the repetitive nail picking. For some patients with multiple involved digits, beginning by daily covering only one finger for two weeks may motivate the individual to treat all the affected nails after they observe improvement. The barrier that can be used include a band aid or hypoallergenic paper tape, protective thermoplastic finger wear and hand wear (such as PLAY Hands), a glove, or a semi-permanent covering (such as an Unna boot which is a gauze bandage that is applied moist to the affected area and subsequently dries to become firm and molded against the skin) [5,6,10,19,20]. 

Close follow up of the individual with onychotillomania has also been suggested. This can be in the form of a weekly manicure which allows not only monitoring of the patient’s progress and an opportunity to provide positive reinforcement, but also the opportunity to apply a topical agent that can cover the nail and/or act as a barrier to prevent splintered cuticles. In addition, the patient should be encouraged to periodically reward themselves (with a special meal or gift) as their nails clinical improve after having stopped or significantly decreased the episodes of nail picking [20]. 

Pharmacotherapy for onychotillomania predominantly includes either selective serotonin reuptake inhibitors or tricyclic antidepressants or antipsychotic medications [14,15]. These agents are mostly effective in nail pickers who have as associated psychiatric condition such as depression [5,6]. However, one group of investigators used N-acetylcysteine (at a daily dose ranging from 1200 to 2400 milligrams) to successfully treated several patients with onychotillomania; they commented that their preference for this agent was based upon superior safety profile compared to other systemic drugs [20].

The patients in this report had several features that were similar to those described in individuals with these nail-associated body-focused repetitive behaviors. Indeed, the nail dystrophy and concurrent dermatodaxia or skin picking were not the issues that prompted them to seek evaluation by the physician; they were incidental findings discovered during their skin examination. The woman was unaware that a habit-tic nail deformity was the cause of her nail dystrophy and was willing to initiate non-pharmacologic treatment by applying paper tape to the affected areas to prevent additional trauma to the proximal nail fold and the underlying nail matrix of her thumbs. In contrast, both men - with either onychophagia or onychotillomania - were cognizant that their nail biting or nail picking was the etiology of their abnormal nail plates; however, neither individual wanted to consider any therapy to decrease or eliminate their nail-focused repetitive behavior.

## Conclusions

Nail-associated body-focused repetitive behavior refers to habit-tic nail deformity, onychophagia, and onychotillomania. Individuals with these nail conditions may concurrently have other body-focused repetitive behaviors affecting their hair and/or skin. Similar to the patients in this report, patients with habit-tic nail deformity are often unaware of the etiology of their abnormal nails whereas nail biters and nail pickers may be cognizant that they are causing their nail dystrophy. Non-pharmacologic and medication-based therapies are available for the management of patients with body-focused repetitive disorders. However, many of the patients with nail-focused repetitive behaviors do not want to initiate any interventions to modify or cease their nail condition. 
